# Factors Associated With Malnutrition in Hospitalized Cancer Patients in a National Oncology Center in Conflict-Affected Settings in Sana’a, Yemen: An Institution-Based Cross-Sectional Study

**DOI:** 10.7759/cureus.45411

**Published:** 2023-09-17

**Authors:** Ahmed H Al-Shahethi, Fares A Mahdi, Emad A Al-Shameri, Fawz M Abol Gaith

**Affiliations:** 1 Department of Community Health and Nutrition, Faculty of Health Sciences, University of Al-Razi, Sana'a, YEM; 2 Department of Community Medicine, Faculty of Medicine, 21 September University of Medical and Applied Sciences, Sana'a, YEM; 3 Department of Nursing, Ibn Alnafis University, Sana'a, YEM

**Keywords:** yemen, sana'a city, cancer, malnutrition, risk factors

## Abstract

Background

Cancer can significantly impact the nutritional status of patients, which can worsen related complications and reduce the effectiveness of treatment. Malnutrition is a common complication among cancer patients, especially among older adults. This study aims to determine the prevalence of malnutrition among hospitalized cancer patients and identify factors associated with malnutrition.

Methodology and methods

The cross-sectional descriptive study was performed on 296 cancer patients admitted to the National Oncology Referral Center in Sana'a, Yemen, between February 1 and March 31, 2022. Malnutrition was diagnosed through clinical assessment and screening tools such as screening tools, body mass index (BMI), weight loss percentage, and mid-upper arm circumference (MUAC). Descriptive statistics, chi-square, and multivariable logistic regression analysis were used to assess factors associated with malnutrition.

Results

Of the 296 patients included in the analysis, most were female 225 (76.0%), and the mean age was 45.8 years. The prevalence of undernutrition, as determined by the BMI score, was 48 (16.2%). The prevalence of malnutrition was higher in females 37 (16.4%) compared to males 11(15.5%). The data indicated that 49% of hospitalized patients had experienced weight loss of 5% or more. Multivariable logistic regression analysis showed that single (AOR=12.93, 95% CI: 1.17-142.77) or widowed (AOR=11.51, 95% CI: 1.05-126.03) marital status, weight loss (AOR=7.56, 95% CI: 3.00-18.69), stomach cancer (AOR=6.77, 95% CI: 1.66-27.70), and breast cancer (AOR=2.60, 95% CI: 1.12-6.03) were associated with an increased risk of malnutrition.

Conclusions

The study highlights the importance of evaluating and addressing the nutritional status of cancer patients, especially at the beginning and during treatment, to improve their clinical outcomes. Prospective studies are recommended to further investigate this issue.

## Introduction

Cancer is a major cause of death worldwide, with more recent estimates of 10 million deaths in 2020. Developing countries face particularly unfavorable outcomes due to limited access to healthcare and increased risk factors such as political and economic instability, as well as lifestyles transitioning towards Western patterns [1]. Surprisingly, almost 20% of cancer patients die due to malnutrition and its related complications, rather than the cancer itself being the main cause of death [2,3].

The Eastern Mediterranean region (EMR) comprises 22 countries, with a population of almost 600 million people, spanning from Morocco in the west to Pakistan in the east. The disease burden in the EMR has changed in the past four decades from primarily communicable diseases to non-communicable diseases, including cancer and cardiovascular diseases [4], similar to many other developing regions. According to GLOBOCAN estimations, the overall age-standardized rate (ASR) of cancer in Yemen in 2020 was 97/100,000 population, with a higher incidence in females at 102.2/100,000 compared to males at 92.7/100,000 [5]. A recent study conducted in Sana'a, Yemen, evaluated the nutritional status of patients with malignant disease based on personal history information and physical examination, and the results showed that 80% of the 60 patients had unintentionally lost weight in the last six months [6].

Due to limited diagnostic and clinical resources, poor quality of medical records, and ongoing civil war for nine years, the accurate incidence of cancer in Yemen is unknown, leading to the ambiguity of the burden of cancer at the national level. Therefore, a national malnutrition for cancer patient registry is required in the Republic of Yemen. The purpose of this study is to determine the prevalence of malnutrition among hospitalized cancer patients and identify factors associated with malnutrition.

## Materials and methods

Study design and setting 

This study is an observational, descriptive cross-sectional study, which was conducted in Sana'a, the capital city of Yemen, between February 1 and March 31, 2022. Sana'a City, the capital city of Yemen is situated in the center of the country at an altitude of 2,300 m. The city has a population of 3,181,655 people [7], and it is growing at a rate of 5.55% per year, accounting for 8.9% of the total population of the Republic of Yemen [8].

The study was performed between February 1 and May 31, 2022, at a National Oncology Referral Center, for oncology in northeastern Yemen, located in Sana’a City of Yemen. Eligible participants included all members of the general population aged 18 and older who were willing to participate in the study. Those patients hospitalized for more than 24 hours, who were fed exclusively through catheter or ostomies for more than 24 hours, with consumptive disorders, or pregnant were excluded from the sample.

The study started after its approval by the Medical Research Committee (MRC) Ethics of the cited Al Razi University number 79, on 15.01.2022.

Sample size and sampling method

The study employed a convenience sampling approach, recruiting participants from the available pool at the time of the study. Specifically, the study participants were recruited from an outpatient clinic located at the National Oncology Center in Sana'a City, Yemen.

The sample size was calculated using the formula

n= (z^2^ *p*(1-p))/e^2^ (1)

where n = sample size, Z = z score (1.96), p = prevalence (80%) [6], and e = margin of error (5%)

n = (1.96)2(0.80)(0.20)/(0.05)2, where n = 246.

To enhance the accuracy of the study and minimize errors due to missing data, a non-response rate of 20% was taken into account. Therefore, the final sample size included 296 participants.

Data collection method

Trained nutritionists employed a convenience sampling approach to recruit participants from an outpatient clinic situated at the National Oncology Center in Sana'a City, Yemen. The participants were assessed through face-to-face interviews, utilizing a structured questionnaire specifically designed to collect the essential information required to fulfill the research objectives. After obtaining informed consent from the patients or their legal guardians, socio-demographic variables, such as age, gender, origin, marital status, occupation, education, and family monthly income, were collected. Behavioral variables, such as smoking, qat chewing, and orange snuff use; nutritional variables, including symptoms of nutritional impact, current weight (kg), habitual weight (kg), height (m), weight loss percentage, previous body mass index (kg/m^2^), and MUAC (cm); clinical variables, such as type of cancer, presence of metastasis, and performance status; and therapeutic variables, such as previous treatment and type of anticancer treatment, were also collected.

To obtain anthropometric variables, such as current weight and height, measurements were taken twice, and if there was a difference between the values, a third measurement was taken, and the average was calculated and recorded as the final value [9].

A fixed vertical digital platform scale with a stadiometer (FILIZOLA™, São Paulo, Brazil) with a variation of 50 g, capacity up to 200 kg, and 0.5 cm precision was used to measure these variables. If it was not possible to measure the stature, it was estimated using the Chumlea equation. The patient's habitual weight (HW) from the past six months was obtained according to their report. The weight loss percentage (%WL) was calculated using the equation [(habitual weight - current weight)/ habitual weight × 100], considering the cutoff point of % WL ≥5 in the past six months. The body mass index (BMI) was obtained by the equation (habitual weight/height2) and was classified according to the recommendations of the World Health Organization (WHO) for adults and the Pan American Health Organization (PAHO)/WHO for the elderly [1]. This variable was stratified into two categories corresponding to underweight (malnourished <18.5) and normal weight (normal weight/overweight).

(% Weight=(usual body weight-actual body weight)/(usual body weight) x 10) (2)

Statistical analysis

To analyze the data, cancers were classified into six groups based on the location of the tumor, including upper gastrointestinal tract, lower gastrointestinal tract, reproductive tract, lung, breast, and other cancers that did not fit into any of the former classifications. Therapeutic variables were stratified into clinical treatment, surgical treatment, hormonal treatment, and palliative treatment. Categorical variables were presented using absolute and relative frequencies, and continuous variables were summarized using mean and standard deviation measures.

Pearson's chi-square test was used for bivariate analysis, and a two-sided test with a significance level of alpha <0.05 was employed. Multivariable logistic regression was used to estimate adjusted odds ratios.

Initially, all variables were included in the analyses, but only variables associated with malnutrition with a p-value <0.25 were retained in the model. The step-wise backward elimination method was applied to develop the final multivariable logistic regression model [10]. A two-sided test with a significance level of alpha <0.05 was used. Statistical Product and Service Solutions (SPSS) software (version 25.0, IBM SPSS Statistics for Windows, Armonk, NY) was used for data analysis.

## Results

Patients’ sociodemographic characteristics

During the study period, a total of 296 cancer patients were admitted, with the majority of the sample being female 225 (76.0%). The mean age of the patients was 45.8±14.5 years, with 151 (51%) falling within the age range of 41-60 years. Most of the participants were married 220 (74.3%), and 137 (46.3%) had no formal education. Among the educated participants, 62 (20.9%) had a primary/intermediate level of education, and six (2%) had a diploma. The highest percentages of respondents were from Dhamar (48, 16.2%), Taiz (41, 13.9%), Ibb (36, 12.2%), and the Capital City Sana'a (35, 11.8%), while the lowest frequencies were from Al-Jawf and Aden (1, 0.3%). Moreover, 125 patients (42.2%) were from urban areas, 91 (30.7%) from rural areas, and 80 (27%) from suburban areas.

The family income of the participants varied from YR 3,500 to YR 1,200,000, with the majority of them belonging to the income group of <YR 50,000 (36.5%). The mean SD monthly household income was YR 82,204.4±100,439.5. Regarding family history of cancer, 82 patients (28%) reported having a family history of cancer, with the father/mother being the most common relative with a history of cancer 29 patients (35%), followed by uncles 27 patients (33%).

Regarding smoking status, 104 patients (35.1%) were smokers/ex-smokers, 172 patients (58.1%) reported no qat chewing, and 123 (41.6%) reported having chewed qat (Table 1).

**Table 1:** Sociodemographic characteristics of the participants.

Variable/Factor	Frequency	Percentage
Gender of patient		
Male	71	24.0
Female	225	76.0
Age of patient (Years)		
< 20 year	12	4.1
20-40	97	32.8
41-60	151	51.0
61-80	34	11.5
> 80	2	0.7
Mean, SD	45.8, 14.5	
Occupation		
Paid work	22	7.4
Farmer	18	6.1
Retirees/benefit	6	2.0
Unemployed	44	14.9
Housewife	186	62.8
Others	20	6.8
Marital status		
Single	23	7.8
Married	220	74.3
Divorced	19	6.4
Widowed	34	11.5
Education levels		
Illiteracy	137	46.3
Read and write	30	10.1
Primary/intermediate	62	20.9
Secondary school	31	10.5
Diploma	6	2.0
University degree or above	30	10.1
Governorate		
Ibb	36	12.2
Sa'adeh	8	2.7
Ad Dali'	3	1.0
Capital city	35	11.8
Dhamar	48	16.2
Ta'izz	41	13.9
Al Mahwit	20	6.8
Amarn	11	3.7
Hajjah	22	7.4
Al Hudaydah	26	8.8
Raymah	11	3.7
Al Bayda'	9	3.0
Abyan	2	0.7
Sana'a Governorate	16	5.4
Lahij	3	1.0
Al Jawf	1	0.3
Hadhramaut	3	1.0
Aden	1	0.3
Permanent residence		
Urban	125	42.2
Suburban	80	27.0
Rural	91	30.7
Family monthly income (YRM)		
< 50,000	108	36.5
50,000-80,000	98	33.1
> 80,000	90	30.4
Mean, SD	82204.4±100439.5	
Family history of cancer		
Yes	82	27.7
No	214	72.3
If yes, who has cancer?		
Grandfather/grandmother	8	9.
Father/mother	29	34.9
Husband/wife	8	9.6
Son/daughter	4	4.8
Uncle	27	32.5
Cousin	4	4.8
Brother	3	3.6
Smoking		
Smoker/ex-smoker	104	35.1
Never smoke	192	64.9
Do you chew qat?		
Yes	123	41.6
No	173	58.3
Nausea		
Yes	154	52.0
No	142	48.0
Loss of appetite		
Yes	129	43.6
No	167	56.4

Sample characteristics according to the types of cancer

The most frequently reported types of cancer, classified by the organic system, were breast cancer 122 (41.2%) and reproductive system cancer (13.2%). Among respondents aged 60 years or older, breast cancer 17 (26.6%) and reproductive system cancer 12 (18.8%) were the most common types. In terms of gender, tumors of the upper gastrointestinal tract were more prevalent among males 16 (22.5%), while breast tumors were more prevalent among females 122 (54.2%).

The sample showed a considerable prevalence of weight loss ≥5% in breast tumors 52 (35.9%), followed by tumors of the upper gastrointestinal tract 22 (15.2%). Low weight was also prevalent among respondents with breast tumors 12 (25.0%) and tumors of the upper gastrointestinal tract 10 (20.8%) (Table 2).

**Table 2:** Sample characteristics according to the types of cancer. ^a^Upper gastrointestinal tract: esophagus, stomach, pancreas, liver, gallbladder, biliary ducts; ^b^Lower gastrointestinal tract: colon, rectum, and anal canal; ^c^Reproductive system: ovarian, cervical, testicular; ^d^Kidney, head and neck, hematological, Hodgkin lymphoma, laryngeal, osteosarcoma, melanoma

Variables		Type of cancer					
	Total sample	UGIT^a^	LGIT^b^	RS^c^	Lung	Breast	Other^d^
		^n (%)^	^n (%)^	^n (%)^	^n (%)^	^n (%)^	^n (%)^
Type cancer- n (%)	296	34 (11.5)	15 (5.1)	39 (13.2)	4 (1.4)	122 (41.2)	82 (27.7)
≥ 60 years- n (%)	64	10 (15.6)	3 (4.7)	12 (18.8)	1 (1.6)	17 (26.6)	34 (53.1)
Gender- n (%)							
Male	71	16 (22.5)	2 (2.8)	9 (12.7)	0 (0.0)	0 (0.0)	44 (62.0)
Female	225	18 (8.0)	13 (5.8)	30 (13.3)	4 (1.8)	122 (54.2)	38 (16.9)
%Weight loss last 6 months (≥ 5), n (%)	145	22 (15.2)	9 (6.2)	8 (5.5)	3 (2.1)	52 (35.9)	51 (35.2)
BMI-low weight- n (%)	48	10 (20.8)	1 (2.1)	4 (8.3)	3 (6.3)	12 (25.0)	18 (37.5)

Chi-square and logistic regression analysis

Association Between Sociodemographic and Influential Factors with Malnutrition

Table 3 displays the distributions of potential risk factors in the malnutrition group and non-malnutrition group. Statistical analysis revealed significant differences in the distributions of age group (p=0.042), gender (p=0.044), presence of chronic disease (p=0.009), smoking status (p=0.041), stomach cancer (p=0.041), and breast cancer (p=0.002) between the two groups.

**Table 3:** Association between sociodemographic and influential factors and malnutrition. ^a^X^2^ – Chi-square; ^b^P*-value <0.05 – Statically significant, ^c^YR – Yemeni Rail, ^¥^chronic disease – diabetic, hypertension, cardiovascular diseases, chronic kidney disease, and depression.

Age group (years)	Total sample (n=296) N (%)	Malnourished (n=48) N (%)	^a^ X^2^	^b^P-value
< 20	12 (4.1)	5 (10.4)	7.4	0.040*
20-30	44 (14.9)	10 (20.8)		
31-40	53 (18.0)	7 (14.6)		
>40	187 (63.2)	26 (54.2)		
Gender			8.1	0.044*
Male	71 (24.0)	11 (22.9)		
Female	225 (76.0)	37 (77.1)		
Occupation			1.2	0.575
Unemployed	70 (23.6)	12 (25.0)		
Paid work	40 (13.5)	4 (8.3)		
Housewife	186 (62.8)	32 (66.7)		
Marital status			9.4	0.019*
Single	23 (7.8)	7 (14.7)		
Married	219 (74.0)	30 (62.5)		
Divorced	19 (6.4)	1 (2.1)		
Widowed	35 (11.8)	10 (20.8)		
Educational levels			5.5	0.337
Illiteracy	137 (46.3)	30 (62.5)		
Read and write	30 (10.1)	4 (8.3)		
Primary/intermediate	62 (20.9)	7 (14.6)		
Secondary school	31 (10.5)	4 (8.3)		
Diploma	6 (2.0)	0 (0.0)		
University degree or above	30 (10.1)	3 (6.3)		
Governorate			2.4	0.224
North region	284 (95.9)	48 (100.0)		
South region	12 (4.1)	0 (0.0)		
Residence			10.8	0.095
Urban	125 (42.2)	20 (41.7)		
Suburban	80 (27.0)	15 (31.3)		
Rural	91 (30.7)	13 (27.1)		
Monthly income (YR)^c^			6.8	0.34
< 50,000	108 (36.5)	24 (50.0)		
50,000-80,000	98 (33.1)	14 (29.2)		
> 80000	90 (30.4)	10 (20.8)		
Family history of cancer			0.2	0.648
Yes	82 (27.7)	12 (25.0)		
No	214 (72.3)	36 (75.0)		
If yes, who has cancer?			4.7	0.262
Grandfather/grandmother	8 (10.6)	3 (25.0)		
Father/mother	29 (38.7)	5 (41.7)		
Husband/wife	8 (10.7)	1 (8.3)		
Son/daughter	4 (5.3)	1 (8.3)		
Uncle	26 (34.7)	2 (16.7)		
Smoking			13.2	0.041*
Smoker	21 (7.1)	1 (2.1)		
Ex-smoker	83 (28.0)	10 (20.8)		
Never smoke	192 (64.9)	37 (77.1)		
Do you chew qat?			0.9	0.321
Yes	124 (41.9)	17 (35.4)		
No	172 (58.1)	31 (64.6)		
Complicated with chronic disease^¥^			11.5	0.009*
Yes	114 (38.5)	10 (20.8)		
No	182 (61.5)	38 (79.2)		
Hypertension			9.46	0.024*
Yes	43 (14.5)	2 (4.2)		
No	253 (85.5)	46 (94.8)		
Cardiovascular disease			8.27	0.041*
Yes	17 (5.7)	1 (2.1)		
No	279 (94.3)	47 (97.9)		
Arthritis			19.27	<0.001*
Yes	50 (16.9)	4 (8.3)		
No	246 (83.1)	44 (91.7)		
Stomach cancer			8.2	0.041*
Yes	15 (5.1)	6 (12.5)		
No	281 (94.9)	42 (87.5)		
Breast cancer			15.2	0.002*
Yes	122 (41.2)	12 (25.0)		
No	174 (58.8)	36 (75.0)		

Association between anthropometric variables and gender

Table 4 displays that 145 (49.0%) of hospitalized patients experienced weight loss with a percentage of ≥5%. The average weight loss percentage score in cancer patients was 11.7±10.1. The results indicated that 49% of cancer patients were malnourished (WL≥5), with the incidence of malnutrition in females 37 (16.4%) significantly higher than in males 11 (15.5%). The BMI of the participants revealed that 132 (44.6%) had a normal BMI, while 48 (16.2%) were underweight. The average BMI percentage score in cancer patients was 24.4±6.9. The incidence of malnutrition in female 37 (16.4%) was significantly higher than in male 11 (15.4%). The average MUAC percentage score in cancer patients was 26.1±4.4, with no significant difference in the incidence of malnutrition between males 10 (14.1%) and females 35 (15.6%).

**Table 4:** Association between anthropometric variables and gender. Reference range of BMI for adults in Yemen: ≥18.5 kg/m^2^; reference range of MUAC (normal ≥21 cm), weight loss in six months (≥10%, kg).

Variable/Factor	N	Mean standard± deviation	Male	Female	P-value
Weight loss percentage	296	11.7±10.1			0.019
0-1.9%	118 (39.9)		28 (39.4)	90 (40.0)	
2-4.9%	33 (11.1)		5 (7.0)	28 (12.4)	
5-9.9%	61(20.6)		8 (11.3)	53 (23.6)	
10-19.9%	55 (18.6)		20 (28.2)	35 (15.6)	
≥20%	29 (9.8)		10 (14.1)	19 (8.4)	
BMI	296	24.4±6.9			0.044
Underweight	48 (16.2)		11 (15.5)	37 (16.4)	
Normal weight	132 (44.6)		40 (56.3)	92 (40.9)	
Overweight	67 (22.6)		15 (21.1)	52 (23.1)	
Obese	49 (16.6)		5 (7.0)	44 (19.6)	
MUAC (cm)	296	26.1±4.4			0.76.3
Normal (≥21 cm)	251 (84.8)		61 (85.9)	190 (84.4)	
Undernourished	45 (15.2)		10 (14.1)	35 (15.6)	

Multivariable logistic regression analysis

The bivariate analysis revealed that age by years, sex, presence of chronic disease, smoking, stomach cancer, and breast cancer were associated with the nutritional status (underweight) of cancer patients. These variables were entered into a multivariable binary logistic regression model to control for the effect of confounding factors.

In the multivariable logistic regression analysis, age (year) was found to be associated with nutritional status, with younger patients having a 96% decreased chance of malnutrition (AOR: 0.96, 95% CI: 0.94-0.99). Patients who were single or widowed were 12.93 and 11.51 times more likely to be malnourished than the reference group of cancer patients (AOR: 12.93, 95% CI: 1.17-142.77), and (AOR: 11.51, 95% CI: 1.05-126.03), respectively.

Patients who experienced weight loss with a percentage of ≥5% were 7.56 times more likely to be malnourished (AOR: 7.56, 95% CI: 3.00-18.69). Finally, patients with stomach and breast cancer were 6.77 times more likely to be malnourished than those with other types of cancer (AOR: 6.77, 95% CI: 1.66-27.70), and patients with breast cancer were 2.60 times more likely to be malnourished than those with other types of cancer (AOR: 2.60, 95% CI: 1.12-6.03) (Table 5).

**Table 5:** Multivariable logistic regression for independent factors associated with malnutrition. B^a^ – coefficient, AOR^b^ – Adjusted odds ratio, 95% ^C^Ic – Confidence interval

Variables	B^a^	AOR^b^	95% CI^c^	P-value
Age (year)	-0.040	0.96	0.94-0.99	0.014
Marital status				
Single	2.56	12.93	1.17-142.77	0.037
Married	1.68	5.38	0.61-47.16	0.129
Divorced	Ref.			
Widowed	2.44	11.51	1.05-126.03	0.045
Weight loss				
Yes	2.020	7.56	3.00-18.69	<0.001
No	Ref.			
Stomach cancer				
Yes	1.91	6.77	1.66-27.70	0.008
No	Ref.			
Breast cancer				
Yes	0.96	2.60	1.12-6.03	0.021
No	Ref.			

## Discussion

Malnutrition of cancer patients is an important but under-recognized problem in Yemen’s healthcare system. The prevalence of malnutrition among cancer patients in this study was (16.2%). The finding of this study was lower than the study done in Vietnam (84.7%) [11], Brazil (33.4%) [12], USA (42%) [13], Thailand (73.3%) [14], and Malaysia (61.9%) [15]. Nevertheless, the results of the present study were close to but slightly lower than those of previous studies conducted in Japan (19.0%) [16] and Korea (22.0%) [17].

The difference in prevalence between the current study and previous studies could be attributed to variations in socio-demographic characteristics, study population, economic status, impaired immune function, and expanded health service provision, as well as the use of different assessment tools. The current prevalence observed in the current study may be due to the side effects associated with chemotherapy, including oral mucositis, intraoral mucositis, intraoral infection, dry mouth, salivary gland inflammation, mucosal bleeding, and intraoral hemorrhage, which can hinder nutritional intake and weaken the immune response. Furthermore, gastrointestinal cancer represents a percentage of 11.5%, which may have hampered their ability to eat.

Additionally, most of the clients in the current study were suffering from gastrointestinal cancer, which could potentially affect their ability to consume food.

Analysis of the percentage of weight loss revealed that 49.0% of the participants experienced a weight loss of more than 5% over the course of six months, which is considered an early sign of malnutrition. In comparison to a study conducted in France, a lower proportion of weight loss was reported, with 17.4%, 12.6%, and 10.9% of the patients experiencing weight loss of 5-10%, 10-15%, and greater than 15%, respectively [3]. A similar study conducted in Ethiopia showed that about 35.8% experienced severe weight loss, and 42.2% experienced moderate weight loss [18].

The presence of these conditions, such as weight loss, is indicative of inadequate nutritional intake due to the interference of the tumor itself on the body's functioning and the side effects of treatment interventions, especially chemotherapy. Additionally, the current findings revealed that 52.0% and 43.6% of the participants experienced nausea and loss of appetite, respectively.

In this study, more than 83.3% of the patients with cancer had comorbidities, such as arthritis, hypertension, diabetes, cardiovascular diseases, chronic kidney disease, and depression. The literature supports the finding that co-prevalence of these conditions is common in cancer patients and can be explained by shared risk factors, including being overweight, having a sedentary lifestyle, smoking, alcoholism, and poor eating habits. It is important to note, however, that chemotherapy can cause changes in an individual's blood pressure and can have a significant impact on the occurrence of hypertension in this population [19,20].

In this study shows that previous or current radiotherapy and chemotherapy were independently associated with an increased risk of malnutrition. This may be due to reduced food intake and/or increased metabolism. Previous studies declared that the association between radiotherapy/chemotherapy and an increased risk of malnutrition may have practical consequences.

Better knowledge of these side effects of chemotherapy-induced and their related chemosensory implications is warranted to generate evidence-based clinical, preventive, and therapeutic guidelines.

In the bivariate analysis age by years, sex, complicated with chronic disease, hypertension, cardiovascular disease, arthritis, smoking, stomach cancer, breast cancer, and lung cancer were associated with nutritional status (underweight) of cancer patients.

The variables mentioned above were entered into a multivariable binary logistic regression model to control the effect of confounders.

Multivariable logistic regression in our study showed that being young reduces the chance of malnutrition by almost 96%. This association was expected, as it is well-known that advanced age predisposes individuals to nutritional deficiencies, particularly in hospitalized patients [21]. In the current study, another significant factor associated with malnutrition was marital status, specifically being single or widowed. This finding is consistent with a study conducted in Indonesia, which found that being unmarried was associated with malnutrition [22].

Our study found that stomach cancer was associated with malnutrition among cancer patients, which is consistent with studies conducted in Latin America [23] and Vietnam [11]. Patients with digestive tract cancer are 23 times more likely to be at nutritional risk compared to other cancer sites, largely due to the catabolic biology of the disease, impaired food intake, absorption of nutrients, and digestion, as well as interruptions in the intestinal passage [24-26].

Furthermore, our study identified that women with breast cancer were significantly associated with malnutrition. This finding is consistent with a previous study [27].

Cancer-induced malnutrition is caused by multiple factors that differ significantly from those associated with simple starvation. These factors include mental health issues, poor food intake, gastrointestinal tract dysfunction, increased energy needs, decreased physical activity, and changes in metabolism in various organs or tissues [28]. Our study has some limitations, including a small sample size and a short duration due to the special conditions being studied. Therefore, it is highly recommended to conduct more studies with longer durations and larger sample sizes to provide more comprehensive and conclusive results.

## Conclusions

The prevalence of malnutrition among adult cancer patients was 16.2%. Malnutrition was higher among female cancer patients than among males. Single or widowed marital status, weight loss, stomach cancer, and breast cancer were found to be significant factors for malnutrition. Therefore, it is crucial to quickly identify individuals with malnutrition or at risk of malnutrition by increasing healthcare professionals' awareness and implementing suitable nutritional screening programs at the beginning and throughout the treatment process. Further qualitative and longitudinal studies are needed to explore the state of malnutrition in cancer patients.
